# Bioactive Polyetheretherketone with Gelatin Hydrogel Leads to Sustained Release of Bone Morphogenetic Protein-2 and Promotes Osteogenic Differentiation

**DOI:** 10.3390/ijms241612741

**Published:** 2023-08-13

**Authors:** Ruonan Zhang, Jun-Ichiro Jo, Ryuhei Kanda, Aki Nishiura, Yoshiya Hashimoto, Naoyuki Matsumoto

**Affiliations:** 1 Department of Orthodontics, Osaka Dental University, 8-1 Kuzuhahanazonocho, Hirakata 573-1121, Osaka, Japan; ruonan-z@cc.osaka-dent.ac.jp (R.Z.); nishiura@cc.osaka-dent.ac.jp (A.N.); naoyuki@cc.osaka-dent.ac.jp (N.M.); 2 Department of Biomaterials, Osaka Dental University, 8-1 Kuzuhahanazonocho, Hirakata 573-1121, Osaka, Japan; yoshiya@cc.osaka-dent.ac.jp; 3 Division of Creative and Integrated Medicine, Advanced Medicine Research Center, Translational Research Institute for Medical Innovation (TRIMI), Osaka Dental University, 8-1 Kuzuhahanazonocho, Hirakata 573-1121, Osaka, Japan; kanda-r@cc.osaka-dent.ac.jp

**Keywords:** Polyetheretherketone (PEEK), gelatin hydrogel, sustained release, bone morphogenetic protein (BMP)-2, osteogenic differentiation, bone tissue engineering

## Abstract

Polyetheretherketone (PEEK) is one of the most promising implant materials for hard tissues due to its similar elastic modulus; however, usage of PEEK is still limited owing to its biological inertness and low osteoconductivity. The objective of the study was to provide PEEK with the ability to sustain the release of growth factors and the osteogenic differentiation of stem cells. The PEEK surface was sandblasted and modified with polydopamine (PDA). Moreover, successful sandblasting and PDA modification of the PEEK surface was confirmed through physicochemical characterization. The gelatin hydrogel was then chemically bound to the PEEK by adding a solution of glutaraldehyde and gelatin to the surface of the PDA-modified PEEK. The binding and degradation of the gelatin hydrogel with PEEK (GPEEK) were confirmed, and the GPEEK mineralization was observed in simulated body fluid. Sustained release of bone morphogenetic protein (BMP)-2 was observed in GPEEK. When cultured on GPEEK with BMP-2, human mesenchymal stem cells (hMSCs) exhibited osteogenic differentiation. We conclude that PEEK with a gelatin hydrogel incorporating BMP-2 is a promising substrate for bone tissue engineering.

## 1. Introduction

The healing of bone defects is a fundamental process in bone tissue reconstruction [1]. However, natural healing rarely occurs when the bony tissue faces extensive defects, unfavorable wound environments, or poor nutritional metabolism [2,3,4]. Large defects, termed critical bone defects, are primarily treated with autografts or allografts [5,6]. However, autografts are limited as they lack a donor supply [7] and pain or bleeding at the donor site [8]. In contrast, allografts are associated with a risk of immune rejection and disease transmission [9,10]. Therefore, the design and preparation of a biomaterial suitable as a bone substitute are required to overcome the limitations of current standard bone transplantation treatments [11].

Polyetheretherketone (PEEK) is a semi-crystalline thermoplastic aromatic polymer extensively employed in tissue engineering as its elastic modulus is comparable to that of human bone. However, the biological inertness and poor bone conductivity of PEEK significantly restrict its clinical applications [12]. To address the issue, various approaches for the surface modification of PEEK have been applied, including bioactive material coatings of hydroxyapatite [13] or titanium dioxide [14], chemical treatments with acid [15] or sulfonation [16], and physical treatments with plasma [17] and ultraviolet irradiation [18]. Furthermore, PEEK has gradually gained attention and widespread applications as a bone replacement implant for craniofacial defect repair, dental implants, joint replacements, spinal cages, and fixation devices [19]. In particular, the incorporation of TiO_2_ into PEEK can enhance its inherent bioactivity, which partially overcomes its biological inertness [20].

The healing process of bone defects is influenced by various factors, such as lack of blood supply, insufficient calcium, phosphorus to facilitate new bone mineralization, and instability at the fracture site [1]. In addition to surface modifications, imparting bone inductivity to PEEK is crucial for the promotion of bone regeneration. Bone morphogenetic protein (BMP)-2 plays a significant role in every stage of bone healing, from the initial phase of fracture repair to later stages [21]. The direct delivery of BMP-2 to the defect site in the solution results in rapid clearance owing to the short half-life of BMP-2 in the body. Thus, to induce bone formation, developing a system for the sustained release of BMP-2 within a specific timeframe is crucial [2]. 

Various degradable and non-degradable biopolymers have been extensively studied as carriers for sustained release [22]. Among biopolymers, gelatin has long been used as a pharmaceutical and biomedical encapsulant owing to its low cost, wide availability, good biocompatibility, and degradability [23]. Gelatin can be chemically modified with various functional groups [24], and gelatin hydrogels can be formed by crosslinking using various methods [25]. Additionally, gelatin hydrogels have been used as carriers for the sustained release of various growth factors, such as hepatocyte growth factor [26], basic fibroblast growth factor [27], and BMP-2 [28]. The carrier system avoids the use of organic solvents, heating, and other harmful operations during the impregnation step of growth factors in the hydrogel, which enables the preservation of their bioactivity [29]. However, the single use of gelatin hydrogels alone may not always be the ideal choice for tissue engineering applications because of their low mechanical strength [30].

The objective of the study was to develop a novel sustained-release system for PEEK by combining it with gelatin hydrogel technology for application in bone tissue engineering. The preparation of PEEK with gelatin hydrogel involved the surface modification of PEEK and chemical binding of the gelatin hydrogel and was characterized by physicochemical methods. The biological properties of PEEK with a gelatin hydrogel incorporating BMP-2 were evaluated in terms of the biodegradation, mineralization, sustained release, and osteogenic differentiation ability of mesenchymal stem cells.

## 2. Results

### 2.1. Preparation and Physicochemical Characterization of PEEK with Gelatin Hydrogel through Polydopamine (PDA) Chemistry

**Figure 1:**
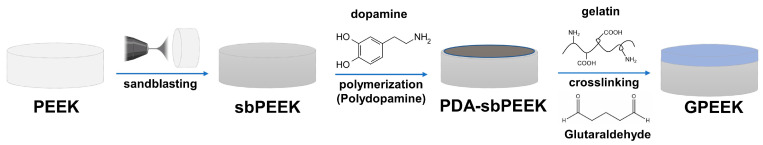
Schematic diagram of procedures for preparation of PEEK with gelatin hydrogel.

**Figure 2:**
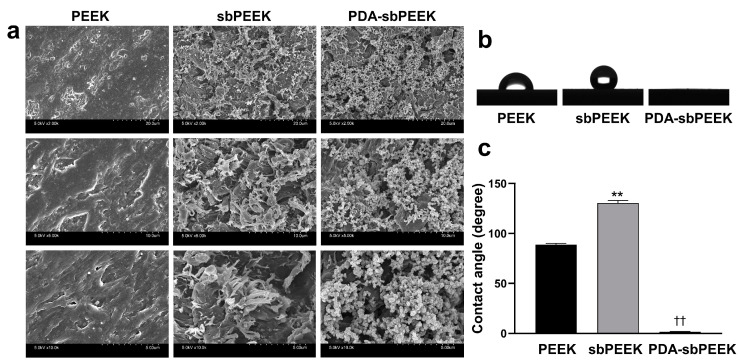
Surface morphology and characteristics of PEEK with or without modifications. (**a**) SEM images of bare PEEK (PEEK), sandblasted PEEK (sbPEEK), and PEEK modified with polydopamine (PDA-sbPEEK). Images are acquired with different magnifications (×2000 (top), ×5000 (middle), and ×10,000 (bottom)). (**b**) Representative images of water droplets on each PEEK substrate. (**c**) Water contact angles of each PEEK substrate. ** *p*  <  0.01; significant against PEEK group. †† *p*  <  0.01; significant against other groups.

The preparation of PEEK with a gelatin hydrogel involved chemically crosslinking gelatin with glutaraldehyde (GA) on the surface of PEEK that was sandblasted and coated with PDA (Figure 1). The PEEK substrates obtained in each step were physicochemically characterized to confirm the success of the preparation procedure. Figure 2a illustrates the scanning electron microscopy (SEM) images of bare PEEK (PEEK), sandblasted PEEK (sbPEEK), and sbPEEK modified with polydopamine (PDA-sbPEEK). The surface of the substrate became rough after sandblasting, whereas some particles that appeared to be PDA were observed without changing the roughness of the PDA-sbPEEK. This tendency is consistent with the results obtained using the roughness meter (Table 1). Water wettability was drastically altered by sandblasting and coating with PDA (Figure 2b). The water contact angle of the substrate increased after sandblasting but decreased after PDA modification (Figure 2c). 

**Figure 3:**
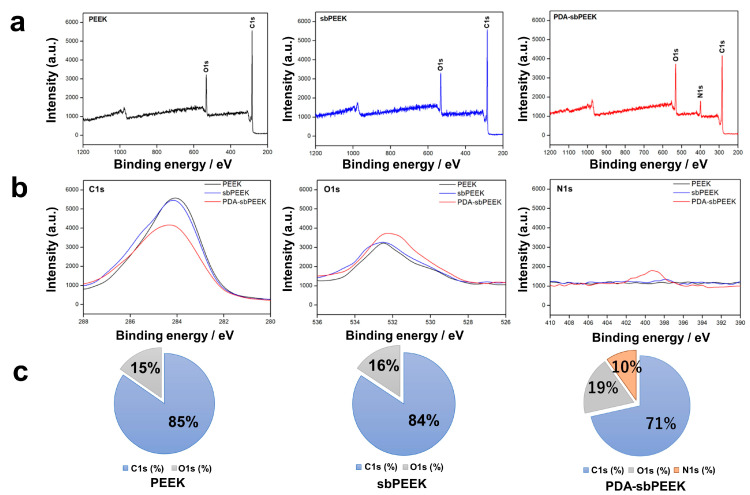
XPS analysis of PEEK, sbPEEK, and PDA-sbPEEK. (**a**) Wide spectra of each PEEK substrate. (**b**) C1s, O1s, and N1s spectra of each PEEK substrate. (**c**) Elemental composition of each PEEK substrate.

To confirm the presence of PDA, X-ray photoelectron spectroscopy (XPS) was performed. The surfaces of PEEK and sbPEEK exhibited carbon (C)1s and oxygen (O)1s peaks, whereas the surface of PDA-sbPEEK exhibited additional nitrogen (N)1s peaks (Figure 3a,b). The elemental compositions of PEEK and sbPEEK were similar. However, a decrease in the percentage of C1s and an increase in the percentages of O1s and N1s were observed on the surface of the PDA-sbPEEK (Figure 3c). The results demonstrate the successful modification of sbPEEK with PDA.

**Figure 4:**
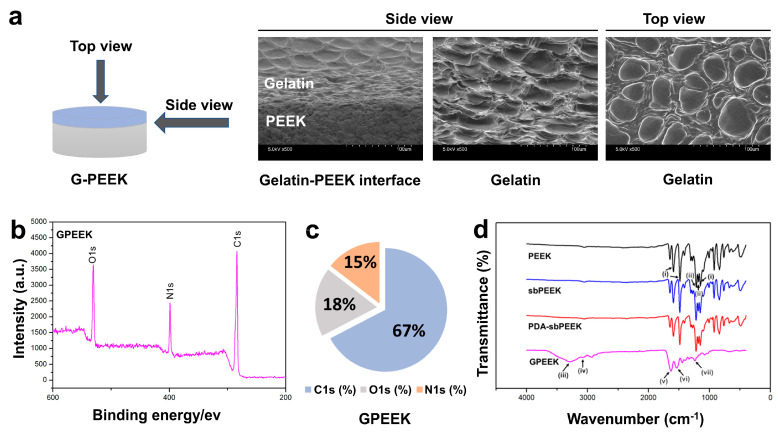
Morphology and chemical properties of PEEK with gelatin hydrogel through PDA chemistry (GPEEK). (**a**) SEM images (×500 magnification) of GPEEK acquired from different directions; interface between gelatin hydrogel and PEEK from side view (left) and gelatin hydrogel with PEEK from side (middle) or top views (right). (**b**) XPS analysis of GPEEK. (**c**) Elemental composition of GPEEK. (**d**) FTIR spectra of PEEK, sbPEEK, PDA-sbPEEK, and GPEEK. Arrows indicate characteristic peaks of (i) phenyl ring, (ii) diphenyl ether groups, (iii) amide A, (iv) amide B, (v) amide I, (vi) amide II, and (vii) amide III.

The morphology and chemical properties of the crosslinked gelatin hydrogel were evaluated to confirm its binding to PDA-sbPEEK (GPEEK). The SEM image from the side view of the interface between the gelatin hydrogel and PEEK reveals tight binding of the gelatin hydrogel to PEEK, while the image from the top view exhibits the presence of gelatin with micropores (Figure 4a). The presence of the gelatin hydrogel on the PEEK was confirmed by XPS and Fourier transform infrared spectroscopy (FTIR) analyses. Moreover, XPS analysis revealed that the GPEEK surface was rich in C, N, and O (Figure 4b,c), which is consistent with the elemental composition of gelatin [31]. Figure 4d demonstrates the FTIR spectra of the PEEK, sbPEEK, PDA-sbPEEK, and GPEEK. The FTIR spectra of all the samples displayed characteristic absorption peaks. The absorption peaks observed at approximately 1150 cm^−1^, 1485 cm^−1^, and 1592 cm^−1^ for PEEK, sbPEEK, and PDA-sbPEEK, respectively, were attributed to the presence of phenyl rings. Peaks at approximately 1183 cm^−1^ and 1219 cm^−1^ were assigned to diphenyl ether groups [32]. The findings indicate that no significant changes in the surface functional groups during the modification process were observed. However, new characteristic absorption peaks were observed for the GPEEK group. Gelatin exhibits absorption bands concentrated in the amide region of its infrared spectrum. The band at approximately 1630 cm^−1^ corresponded to amide I (representing the C=O stretching vibration/hydrogen bonding with COO-). The amide II band at 1537 cm^−1^ is attributed to the bending vibration of the N-H groups and the stretching vibration of the C-N groups. The presence of the amide III bands (indicating in-plane vibrations of C-N and N-H groups involved in amide bonding or vibrations of glycine CH_2_ groups) is prominent at 1238 cm^−1^. Furthermore, the amide-A peak (representing NH- stretching coupled with hydrogen bonding) is observed at 3284 cm^−1^, and the amide-B peak (corresponding to the asymmetric stretching vibrations of =C-H and -NH_3_^+^) is evident at 3078 cm^−1^. The appearance of the absorption peaks aligns with the structural characteristics of gelatin, confirming the successful binding of gelatin to the surface of PEEK [33].

**Figure 5:**
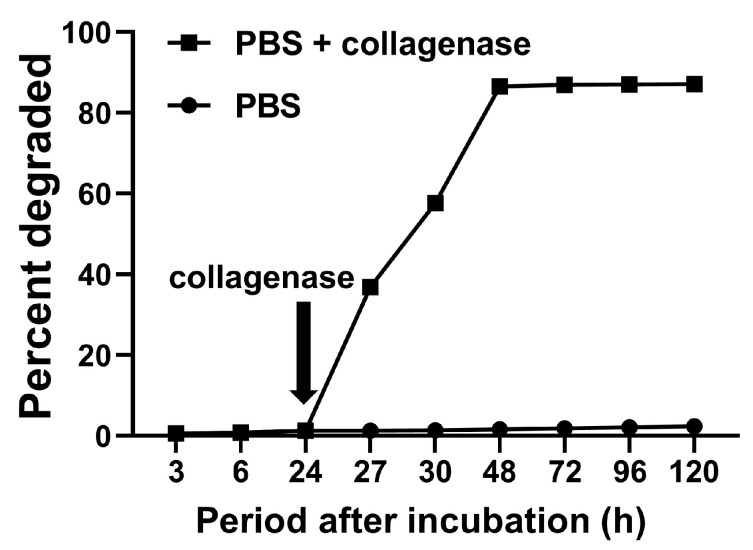
Degradation profiles of gelatin hydrogel bound on PEEK in PBS (●) or PBS with collagenase (■). Collagenase (25 μg/mL) was added at each time point from 24 h after incubation in the PBS + collagenase group.

Figure 5 demonstrates the degradation profiles of the gelatin hydrogels bound to PEEK. The gelatin hydrogel hardly degraded in phosphate-buffered saline (PBS), whereas the degradation of the gelatin hydrogel was accelerated in PBS containing collagenase.

### 2.2. Biological Potentials of GPEEK

**Figure 6:**
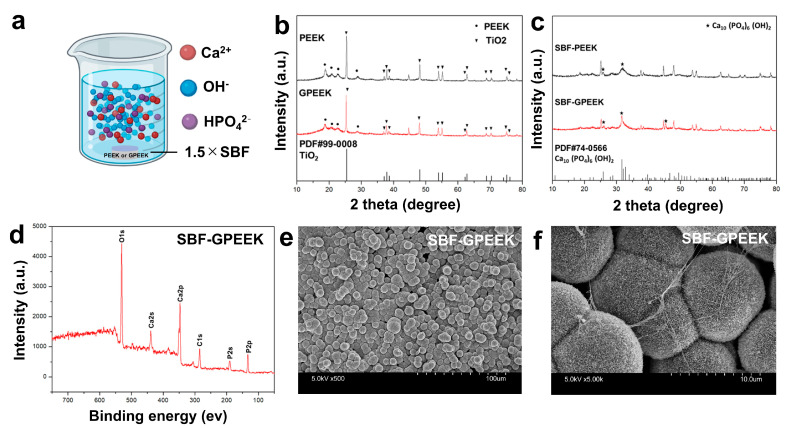
In vitro assessment of mineralization potential in the PEEK or GPEEK by immersion in the simulated body fluid (SBF) with 1.5 times concentration. (**a**) Schematic diagram of experiment. (**b**) XRD analysis of PEEK and GPEEK. (**c**) XRD analysis of PEEK and GPEEK after SBF immersion (SBF-PEEK and SBF-GPEEK). The symbol # indicates the number of the standard card for XRD. (**d**) XPS analysis of SBF-GPEEK. (**e**,**f**) SEM images of SBF-GPEEK with magnifications of ×500 (**e**) and ×5000 (**f**).

The mineralization potential of GPEEK was assessed in vitro by immersion in the simulated body fluid (SBF) with 1.5 times concentration (1.5 ×SBF) (Figure 6a). Several methods were used to characterize the degree of mineralization after immersion in SBF. The X-ray diffraction (XRD) patterns of PEEK and GPEEK before SBF immersion exhibited the characteristic peaks of titanium dioxide. This is because titanium dioxide is present in the medical-grade PEEK used in this study (Figure 6b). The XRD patterns of PEEK and GPEEK after SBF immersion (SBF-PEEK and SBF-GPEEK) demonstrated the characteristic peaks of hydroxyapatite (Figure 6c). The shape of the peak near 32° for SBF-GPEEK was sharper than that for SBF-PEEK, suggesting that the mineralization potential of GPEEK was higher than that of PEEK. Moreover, XPS analysis of SBF-GPEEK confirmed the presence of calcium, phosphorus, and O, which are the key components of hydroxyapatite (Figure 6d). In addition, a spherical needle-like shape resembling hydroxyapatite was observed for SBF-GPEEK using SEM (Figure 6e,f).

**Figure 7:**
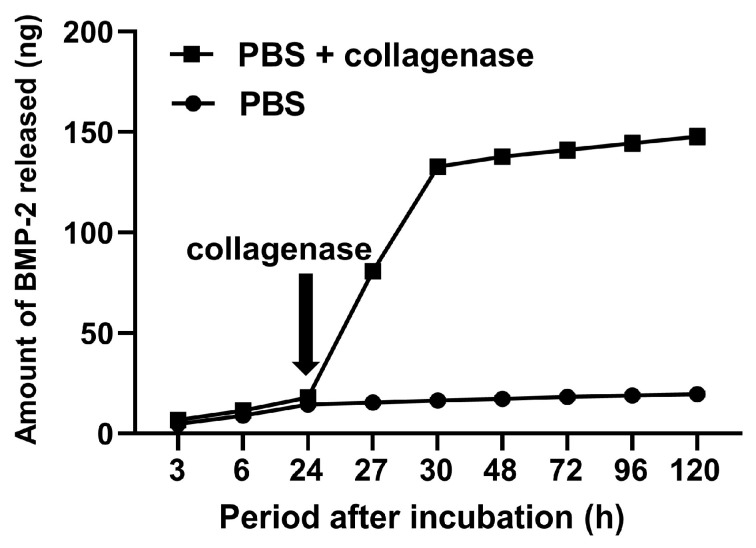
Release profiles of BMP-2 from gelatin hydrogel bound on PEEK in (●) or PBS with collagenase (■). Collagenase (25 μg/mL) was added at each time point from 24 h after incubation in the PBS + collagenase group.

To explore the sustained-release ability of GPEEK, the release profiles of BMP-2 were evaluated by incubation in PBS with or without collagenase (Figure 7). In PBS, BMP-2 was gradually released over time; however, the release rate was slow. In contrast, the rate of BMP-2 release from the GPEEK cells was drastically accelerated by the addition of collagenase.

### 2.3. Osteogenic Differentiation of Stem Cells on PEEK Substrates

**Figure 8:**
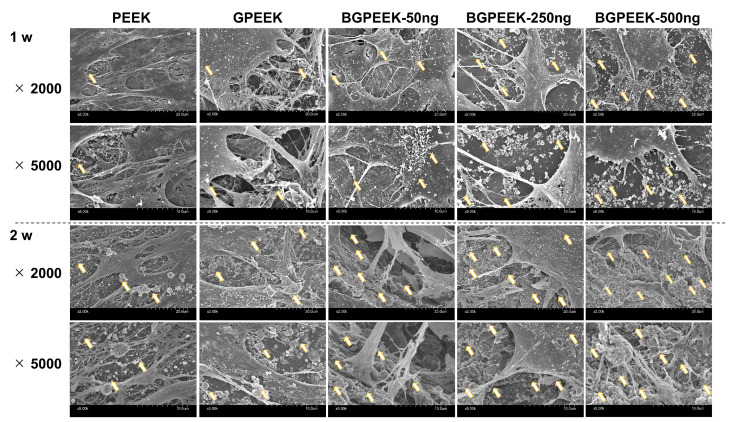
SEM images (magnification; ×2000 or 5000) of hMSCs cultured on PEEK, GPEEK, and GPEEK incorporating different amounts of BMP-2 (50, 250, or 500 ng) under the induction of osteogenic differentiation for 1 or 2 weeks. Yellow arrows indicate the mineral secretion and deposition from the cells of osteogenic differentiation.

To investigate the feasibility of using PEEK with sustained-release and osteogenic differentiation abilities, human mesenchymal stem cells (hMSCs) were cultured on different PEEK substrates under induced osteogenic differentiation. The SEM image (Figure 8) illustrated that the pseudopodia of the cells tightly adhered to the PEEK substrates, irrespective of gelatin binding and BMP-2 incorporation. In addition, the deposition of some mineral-like particulate substances was observed, and the number of depositions increased with an increase in the concentration of BMP-2 and culture time.

**Figure 9:**
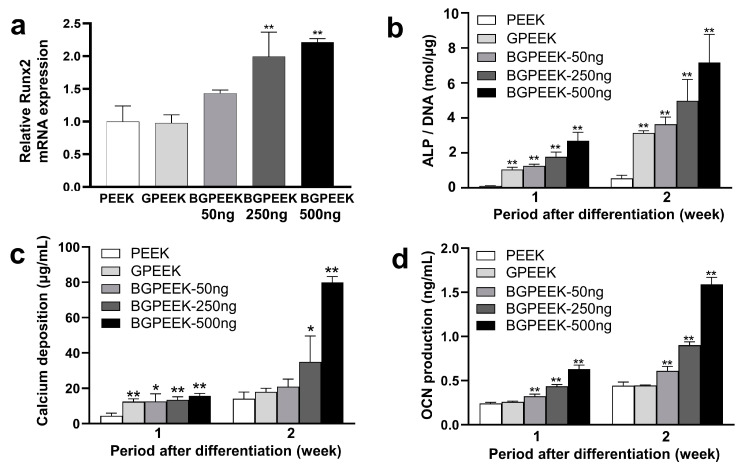
Evaluation of osteogenic differentiation for hMSCs cultured on PEEK, GPEEK, and GPEEK incorporating different amounts of BMP-2 (50, 250, or 500 ng) under induced osteogenic differentiation. (**a**) Level of expression of Runx2 mRNA for hMSCs 3 days after induction of osteogenic differentiation. ALP activity (**b**), calcium deposition (**c**), and osteocalcin production for hMSCs 1 or 2 weeks after induction of osteogenic differentiation (**d**). * *p* < 0.05, ** *p* < 0.01; significant against PEEK group.

The osteogenic differentiation behavior of hMSCs cultured on the different PEEK substrates was evaluated using several methods (Figure 9). Runt-related transcription factor 2 (Runx2) messenger ribonucleic acid (RNA) expression was strongly associated with an increase in the BMP-2 amount incorporated into the GPEEK (Figure 9a). Similarly, the production of alkaline phosphatase (ALP), calcium deposition, and osteocalcin (OCN) increased with an increase in the BMP-2 amount incorporated into GPEEK, as well as during the osteogenic differentiation culture (Figure 9b–d).

## 3. Discussion

This present study demonstrated the feasibility of using GPEEK as a carrier for the sustained release of BMP-2 and as a culture substrate for the osteogenic differentiation of hMSCs. Several studies have been conducted on PEEK with hydrogels for biomedical applications. In the aforementioned studies, the main objectives of hydrogel binding to PEEK include the improvement of water wettability and lubrication [34,35] and the incorporation of drugs to provide antibacterial activity and bone regeneration [36,37,38,39]. Most hydrogels bound to PEEK demonstrate low biodegradability in the body, which may interfere with the function of PEEK itself in the case of long-term usage. In this context, this is the first report on the preparation of PEEK with biodegradable hydrogels that achieved a sustained release of biofunctional factors.

Various biomolecular modification methods for PEEK have been reported [40], including physical adsorption and direct covalent bonding through silanization [41,42]; selective reduction, ester formation [43,44], and oxidation; and amide formation through carbodiimide chemistry [45,46]. The PDA modification method used in this present study is the third method used to bind biomolecules to PEEK. When added to PEEK at alkaline pH, dopamine is polymerized and attached to provide reactive functional groups (primary amino and catechol/quinone groups) on the PEEK surface [47,48]. Before PDA modification, the PEEK was sandblasted with alumina particles. Sandblasting is a technique commonly used in the surface treatment of implants to remove surface impurities and increase roughness, thereby improving the binding strength between the substances to be attached [49]. In the present study, sandblasting increased the roughness of PEEK (Table 1), which is consistent with previous research results performed by other research groups [50]. The PDA modification of PEEK was also confirmed by the results of the XPS analysis (Figure 3) and contact angle measurements (Figure 2b,c).

In this study, we prepared GPEEK by adding a solution of gelatin and GA to PDA-modified sandblasted PEEK (PDA-sbPEEK). The binding of the gelatin hydrogel and PDA-sbPEEK was likely achieved via two chemical mechanisms based on PDA and GA. The catechol/quinone groups in PDA reacted with the amine groups of the gelatin to form covalent bonds via Michael addition or Schiff base reactions [51]. The aldehyde groups in GA react with the amine groups of gelatin and PDA to crosslink the gelatin and PDA chains. Successful binding of the gelatin hydrogel to PDA-sbPEEK was confirmed morphologically by SEM (Figure 4a) and physicochemically by XPS (Figure 4b,c) and FTIR (Figure 4d).

The degradation and mineralization behaviors of GPEEK were investigated to confirm its biological activity. The PEEK-bound gelatin hydrogel was degraded in PBS containing collagenase (Figure 5), and the behavior is consistent with that of the gelatin hydrogel without any substrate [52]. The mineralization behavior of GPEEK was evaluated by soaking in a 1.5 × SBF solution [53]. Additionally, XRD analysis revealed that the characteristic peaks of hydroxyapatite were observed in PEEK even without binding to the gelatin hydrogel (Figure 6c), which was due to the presence of titanium dioxide on the surface of the medical-grade PEEK to enhance the inherent bioactivity [20]. However, under the same conditions, the degree of crystallinity of the mineralized substance from GPEEK after soaking in the 1.5 × SBF solution was higher than that from PEEK (Figure 6c). This is because the negatively charged carboxylic acid groups on the surface of the gelatin hydrogel attracted calcium ions in the 1.5 × SBF solution and promoted hydroxyapatite nucleation by further adsorption of phosphate and calcium ions [54]. Considering that improved crystallinity contributes to improved osseointegration [55], GPEEK is a promising material for implants that require bonding to the host bone.

Sustained-release systems have attracted extensive research attention for many biomedical and tissue engineering applications that enhance drug targeting, improve pharmacokinetics, and reduce toxicity, resulting in significant improvements over conventional therapies. Gelatin hydrogel is one of the most widely used hydrogels for the sustained release of drugs, including growth factors [56], genes [57], and low-molecular-weight drugs [58]. Several studies have demonstrated that the release of drugs from hydrogels is accompanied by their degradation [59,60,61]. In this study, the release behavior of BMP-2 from GPEEK was explored to confirm its sustained-release capability. The release of BMP-2 from the GPEEK in PBS was gradual, whereas its release was accelerated in PBS containing collagenase (Figure 7). This was due to the degradation of the hydrogel by collagenase (Figure 5), suggesting that the release profile of BMP-2 correlated with hydrogel degradation, as previously reported [60].

Moreover, BMP-2, a member of the transforming growth factor (TGF)-β superfamily, is known for its roles in bone and cartilage development, regeneration, and repair [21]. In addition, BMP-2 exhibits potent osteoinductive properties that are crucial for bone formation by stimulating the differentiation of MSCs into osteoblasts [2]. In the present study, osteogenic differentiation was observed in hMSCs cultured on GPEEK containing BMP-2, and the extent of osteogenic differentiation increased with an increase in the amount of BMP-2 incorporated (Figure 8 and Figure 9). The continuous action of BMP-2 released from GPEEK on hMSCs results in enhanced osteogenic differentiation. The result strongly indicates that BMP-2 released from GPEEK retains its biological activity to induce the osteogenic differentiation of hMSCs. Therefore, the potential of the GPEEK system to achieve the sustained release of BMP-2 holds significant promise for applications in tissue engineering and regenerative medicine.

The present study has several limitations. The GPEEK system should be used for in vivo experiments to confirm its feasibility. Considering the usage of the GPEEK system as a scaffold for bone tissue engineering, the processing of three-dimensional porous PEEK to induce stem cell invasion inside and vascularization [62] will be required. In addition, the preparation procedures developed in the present study should be applied to create GPEEK with different types of gelatin and biofunctional molecules to achieve versatility and practicality. The aforementioned issues should be addressed in future studies. 

## 4. Materials and Methods

### 4.1. Modification of PEEK with PDA

Cylindrical PEEK (medical-grade, Ketron LSG CLASSIX) with a diameter of 10 mm and height of 3 mm was purchased from Yasojima Proceed Co. Ltd., Kobe, Japan. The PEEK surface was sandblasted using JET BLAST II (J. MORITA Corp., Osaka, Japan) with brown fused alumina particles as the grinding material (SBT F180, A-43, Resonac Holdings Corp., Tokyo, Japan). Sandblasting was performed manually using a handle to ensure uniformity. Subsequently, the sandblasted PEEK was thoroughly rinsed using a high-pressure washing machine (JS-2500s, J. MORITA Corp.) to remove any residual sand and obtain PEEK with a rough surface (sbPEEK). After ultrasonic cleaning with acetone, ethanol, and deionized water, sbPEEK was immersed in a solution (2 mg/mL) of dopamine hydrochloride (Sigma-Aldrich, St. Louis, MO, USA) dissolved in 10 mM Tris-HCl (pH 8.5, FUJIFILM Wako Pure Chemical Corp., Osaka, Japan) and placed on a rotating table at 37 °C for 24 h for attachment and polymerization on sbPEEK. The reacted sbPEEK was washed three times with distilled water and dried to obtain PDA-sbPEEK. 

The surface morphologies and microstructures of the PEEK substrates were examined using SEM (S-4800; Hitachi, Ltd., Tokyo, Japan). Before SEM imaging, each sample was coated with a thin conductive osmium layer using an osmium coater (HPC-20; VACUUM DEVICE Co. Ltd., Ibaraki, Japan). The wettability of each PEEK substrate to water was measured at room temperature by adding distilled water droplets (0.7 µL) onto each substrate using a contact angle meter (LSE-ME2; NiCK Corp., Saitama, Japan). The surface roughness of each PEEK substrate was measured using a surface-roughness test instrument (DR130, SATO TEC. Corp., Shizuoka, Japan). Surface elemental analysis of each PEEK substrate was performed using XPS (ESCA-5600; ULVAC-PHI, Inc., Kanagawa, Japan), and the elemental composition was calculated based on the analysis results. 

### 4.2. Binding of Gelatin Hydrogel onto PDA-sbPEEK

Gelatin (isoelectric point 9.0) was supplied by Nitta Gelatin Co., Ltd., Osaka, Japan. Furthermore, GA (25% in water, Nacalai Tesque, Inc., Kyoto, Japan) was added as a chemical crosslinker to the gelatin aqueous solution (5 wt%) at a solution volume ratio of 1:500, and the mixture was immediately dropped onto PDA-sbPEEK at a solution volume ratio to PEEK surface area of 1.0 (μL/mm^2^). After the crosslinking reaction overnight at 4 °C, the hydrogel was reacted with 100 mM glycine aqueous solution for 1 h at room temperature to block any residual aldehyde groups of GA and then washed with double-distilled water (DDW) three times to obtain GPEEK. Additionally, GPEEK was freeze-dried (FDU-200, EYELA Co., Ltd., Tokyo, Japan) and stored until use.

The surface morphology of GPEEK and the microstructure of the interface between gelatin and PEEK in GPEEK were observed by SEM (S-4800) using the same procedure described above. Surface elemental analysis of GPEEK was carried out by XPS (ESCA-5600) using the procedure described above. To identify the chemical bonds on the surface of each PEEK substrate, FTIR spectroscopy (IRAffinity-1S; SHIMADZU Corp., Kyoto, Japan) was used.

### 4.3. Degradation Behavior of Gelatin Hydrogel in GPEEK

After GPEEK was immersed in PBS, the supernatant was collected at 3, 6, 24, 27, 30, 48, 72, 96, and 120 h in one group. In the other group, 24 h after immersion, PBS was changed to PBS with calcium and magnesium ions containing collagenase D (25 µg/mL), and the supernatant was subsequently collected, and the fresh collagenase solution was added at 27, 30, 48, 72, 96, and 120 h. The amount of degraded gelatin fragments contained in the supernatant was measured using the Pierce™ micro Bicinchoninic acid Protein Assay Kit (Thermo Fisher Scientific Inc., Waltham, MA, USA) according to the manufacturer’s instruction.

### 4.4. In Vitro Mineralization Assay for PEEK Substrates

Each PEEK substrate (PEEK or GPEEK) was immersed in SBF with 1.5 times concentration (1.5 × SBF) [63] for 1 week, followed by gentle rinsing with DDW. The crystal structure of the substance deposited on the surface of each substrate was evaluated using the XRD system (XRD-6000, SHIMADZU Corp., Kyoto, Japan) with Cu Kα radiation at 40 kV and 30 mA. The scanning speed and 2θ range were set to 2°/min and 10–80°, respectively.

### 4.5. Incorporation of Bone Morphogenetic Protein (BMP)-2 into GPEEK

PBS (pH 7.4) containing various amounts of BMP-2 (50, 250, and 500 ng; R&D Systems, Inc., Minneapolis, MN, USA) was dropped onto freeze-dried GPEEK and incubated for 1 h at 37 °C to obtain GPEEK with incorporated BMP-2 (BGPEEK). The BGPEEK-x ng indicated the GPEEK-incorporating x ng of BMP-2. The BGPEEK-500 ng was soaked in 500 µL of PBS. The supernatant was collected after incubation in PBS or PBS containing collagenase D using the procedures described in Section 4.2. The amount of released BMP-2 into the supernatant was measured using a Quantikine^®^ ELISA kit for BMP-2 (R&D Systems, Inc., Minneapolis, MN, USA) according to the manufacturer’s instructions.

### 4.6. Osteogenic Differentiation for Human Mesenchymal Stem Cells Cultured on Various PEEK Substrates

For the culture of hMSCs (Lonza Group Ltd., Basel, Switzerland), MSCGM™ Mesenchymal Stem Cell Growth Medium BulletKit™ (Lonza Bioscience, Basel, Switzerland) was used. The cells were cultured in a regular medium, which was changed every 3 days. Upon reaching subconfluence, the cells were harvested and subcultured. Cells from passages three to five were used in subsequent experiments. Moreover, hMSCs (40,000 cells) were seeded on each PEEK substrate and cultured with alpha-minimum essential medium (α-MEM, Nacalai Tesque Inc., Kyoto, Japan) containing 10% fetal bovine serum (FBS, Thermo Fisher Scientific Inc.), 50 mM l-ascorbic acid 2-phosphate (Nacalai Tesque Inc., Kyoto, Japan), 10 mM *β*-glycerophosphate (FUJIFILM Wako Pure Chemical Corp.), and 10 nM dexamethasone (Nacalai Tesque Inc., Kyoto, Japan). The extent of osteogenic differentiation of hMSCs cultured on each PEEK substrate was investigated using the following evaluations: Total RNA from hMSCs cultured on each PEEK substrate for 3 days of osteogenic differentiation was extracted using RNeasy Mini Kit (QIAGEN, Venlo, Netherlands). The extracted RNA was reverse-transcribed using SuperScript IV VILO Master Mix (Thermo Fisher Scientific Inc., Waltham, MA, USA). To quantify the expression of Runx2 (Hs01047973_m1) and glyceraldehyde-3-phosphate dehydrogenase GAPDH (4310884E), a TaqMan gene expression assay from Thermo Fisher Scientific in conjunction with the QuantStudio 3™ PCR System from Applied Biosystems (Thermo Fisher Scientific Inc., Waltham, MA, USA) was used. Gene expression results from the negative control group were analyzed using the ΔΔCt method. The data were normalized to GAPDH and the relative gene expression for each group was calculated accordingly. The experiment was repeated thrice for each group.

Cells cultured on each PEEK substrate for 7 and 14 days of osteogenicdifferentiation were treated with 0.2% Triton X-100 (Nacalai Tesque, Inc., Kyoto, Japan) to extract the cellular components. Then, the ALP activity was quantified using a One-Step PNPP™ assay (Thermo Fisher Scientific Inc., Waltham, MA, USA). The absorbance was measured at 450 nm using a microplate reader (SpectraMax iD3; Molecular Devices, San Jose, CA, USA). The activity was normalized to the DNA content determined using the PicoGreen dsDNA assay kit (Thermo Fisher Scientific Inc., Waltham, MA, USA).

After the extraction of components of hMSCs by the same procedure for the ALP assay described above, the extracellular calcium was dissolved in 10% formic acid (Nacalai Tesque Inc., Kyoto, Japan) and measured using a Calcium E-test kit (FUJIFILM Wako Pure Chemical Corporation) in accordance with the manufacturer’s guidelines.

The supernatants of hMSCs cultured on each PEEK substrate were collected after 7 and 14 days of osteogenic differentiation. The OCN concentration in the supernatant was measured using the Human Gla-Osteocalcin High-Sensitive EIA Kit (Takara Bio Inc., Shiga, Japan) according to the manufacturer’s instructions.

### 4.7. Statistical Analysis

Data were analyzed and presented as the mean ± standard deviation. Parametric data were analyzed using a one-way analysis of variance with Tukey’s test. Statistical analyses were performed using GraphPad Prism ver. 8 (GraphPad Inc., Boston, MA, USA).

## 5. Conclusions

A novel sustained-release system for BMP-2 from PEEK was developed by chemically binding a gelatin hydrogel using PDA chemistry. Moreover, GPEEK exhibited mineralization potential in SBF. The osteogenic differentiation of hMSCs was promoted by the sustained release of BMP-2 from GPEEK. Thus, GPEEK is a feasible bioactive implant material for bone tissue engineering.

## Figures and Tables

**Table 1:** Surface roughness of each PEEK substrate.

Substrate	Roughness (μm)
PEEK	0.67 ± 0.01
sbPEEK	0.82 ± 0.04
PDA-sbPEEK	0.85 ± 0.03

## Data Availability

Not applicable.
